# Comparison of cost of care in patients with flares vs. remission in ulcerative colitis: A perspective from a developing country

**DOI:** 10.3892/mi.2025.274

**Published:** 2025-10-03

**Authors:** Ashutosh I. Yadav, Vishavdeep Singh Rana, Amol N. Patil, Parna Pathak, Usha Dutta, Vishal Sharma

**Affiliations:** 1 Department of Gastroenterology, Postgraduate Institute of Medical Education and Research, Chandigarh 160012, India; 2 Department of Internal Medicine, Postgraduate Institute of Medical Education and Research, Chandigarh 160012, India; 3 Department of Pharmacology, Postgraduate Institute of Medical Education and Research, Chandigarh 160012, India

**Keywords:** inflammatory bowel disease, ulcerative colitis, cost of illness, pharmacoeconomics

## Abstract

Ulcerative colitis (UC) is a chronic relapsing-remitting disease that results in not only physical, mental and social, but also a significant financial burden to patients and their caregivers. The present study aimed to analyse the monthly cost of care for patients with UC in remission during their regular follow-up and compare this to the expenses born during an episode of acute exacerbation in order to estimate the costs of such episodes of acute severe UC (ASUC). Patients in remission defined using the simple clinical colitis activity index (SCCAI) of <3 were recruited from the outpatient department. Patients with flares were those requiring admission for ASUC. Demographic and clinical data were recorded for each patient. A prevalence-based, micro-costing, human capital approach was used to estimate the direct and indirect monthly per capita mean cost-of-illness. The present study enrolled 25 patients with UC who were in remission (SCCAI of <3) and 51 patients with UC who presented with flares and required hospitalisation for ASUC between January, 2022 to June, 2024. The average monthly cost of care for patients with UC in remission in a tertiary government setup was calculated to be 4,112/- INR. The direct medical cost was 3,676/- INR and the direct non-medical cost was 435/- INR. The cost of management of an episode of ASUC was 44,634/- INR per individual per episode. The direct medical cost was 20,038/- INR, the direct non-medical cost was 4,087/- INR and the indirect cost was 20,509/- INR. The cost of the flares managed surgically was 155,967±100,554/- INR, which was significantly higher than that of flares managed medically (26,922±22,751/- INR; P<0.001). The cost of illness during episodes of acute flares contribute disproportionately to a high financial burden in care of patients with UC. Direct medical costs due to the cost of medications is a major contributor to the cost of care for patients with UC in remission whereas during a period of flares, the indirect cost due to productivity loss is responsible for the majority of the financial burden.

## Introduction

Chronic disease, including inflammatory bowel disease (IBD) is associated with significant costs. Although a number of studies have evaluated the costs of care (including medication, hospitalisation and surgery ambulatory visits) in the high-income regions, the data are relatively limited in the lower- and middle-income countries (LMICs) (1). The care of patients with IBD in LMICs has certain additional caveats; the use of advanced therapies including biologicals and small molecules is limited by a lack of access, availability and comfort with their use (2,3). There may also be an additional role of risk of infections, including tuberculosis and the higher uptake of complementary and alternative medication that may reduce the compliance with standard therapies, resulting in increased flares (4).

The treatment paradigm for ulcerative colitis (UC), a chronic relapsing-remitting disease, continues to evolve and the data for a top-down approach for UC are limited, as are those against Crohn's disease (5). Therefore, standard therapies, including 5-aminosalicylates and thiopurines remain the major tools in the treatment of UC, particularly in LMICs (6). IBD, including UC results in not only in a physical, mental and social burden, but is also associated with a marked financial burden to patients, as well as their caregivers (7). Any flare of the disease increases this burden. The need for rescue therapy in the form of advanced therapies (anti-TNF agents, anti-integrins, anti-IL-12/23 and small molecules) or surgery increases both the short-, as well as long-term financial burden. However, the selection of therapy in numerous regions of the globe is dictated by cost considerations, and 5-aminosalicylic acid (5-ASA) compounds and thiopurines are often used as a first-line therapy; surgery appears fairly early in the treatment paradigm. It is unclear whether the cost of flares is substantially higher than ongoing therapy during periods of remission in LMICs (8). The present study aimed to analyse the monthly cost of care of patients with UC in remission during their regular follow-up and compare this to the expenses born during an episode of acute exacerbation, in order to estimate the costs of such episodes of flares. Cost-of-illness analysis for patients with UC in remission, as well as for those with acute exacerbations was performed; this included direct costs (medical and non-medical), as well as indirect costs during out-patient department (OPD) visits and in-patient admissions.

## Patients and methods

### Study setting

The present study was a prospective observational, cross-sectional, single-centre study conducted between January, 2022 to June, 2024; the study included 25 patients with UC who were in remission [simple clinical colitis activity index (SCCAI) score <3] and 51 patients with UC who presented with flares and required hospitalisation for acute severe ulcerative colitis (ASUC) at the Post Graduate Institute of Medical Education and Research (PGIMER) in Chandigarh, India. The present study was approved by the Institute Ethics Committee of the Post Graduate Institute of Medical Education and Research (INT/IEC/2022/000488). A written informed consent/assent was obtained from all subjects or their legal guardians.

### Study population. UC cases in remission

UC cases in remission were managed in the OPD. Patients were deemed to be in disease remission with both i) a SCCAI score <3 with a stable bowel frequency over the past 6 months; and ii) a normal faecal calprotectin level (<100 mcg/g) (4). The evaluation of patients included an investigation of clinical history and an examination, periodic biochemical testing, faecal calprotectin level assessment and an unprepared sigmoidoscopic examination. Any treatment modification was determined as per the disease activity during OPD visits. The total duration for OPD follow-up for UC cases in remission was 6 months.

*Patients with flares requiring hospitalisation*. Those patients who had severe flares requiring hospital admission were included and the majority of these were patients had ASUC. Patients who were admitted for non-flare conditions, such as intercurrent infection were excluded. The evaluation of cases with flares included an investigation of clinical history and an examination, biochemical testing, stool analysis for routine examination, faecal calprotectin level assessment, *Clostridioides difficile* toxin assay and unprepared sigmoidoscopic examination. Standard treatment for ASUC was with intravenous steroid and venous thromboprophylaxis, and 5-aminosalicylate optimisation was performed, followed by a response assessment using the Oxford criteria and those deemed to be non-responsive were offered second-line therapy (intravenous cyclosporine or intravenous infliximab or colectomy) (9).

### Cost estimation

The data on expenses were categorised as direct medical, direct non-medical and indirect costs, which were summated as total expenditures. The cost of treatment was defined as follows: i) Direct medical costs: These included the cost of drugs, as well as any investigations, including the cost of surgery if required. The cost of drugs was calculated by taking the average cost of five commonly used drug brands used for treatment which were available locally, while the cost of investigations included the standard cost of tests, as fixed by the institution. ii) Direct non-medical costs: These included the cost of stay and travel. The cost of stay included the cost of in-hospital admission, as well as the per-day-cost of admission during the admitted period for patients, as well as the stay of lodging for 1 patient attendant. Travelling costs included standard railway ticket costs for sleeper class, as fixed by the government railway agency (IRCTC) from Chandigarh to the nearest railway station of the hometown of the patient. iii) Indirect costs: These included total job earnings lost during the treatment period. These included daily wages lost for patients, as well as the attendant of each patient.

### Statistical analysis

All descriptive and inferential statistics were generated using SPSS software version 23 (IBM Corp.). Cost data are expressed as the average per capita monthly cost in Indian rupees (INR). Wherever appropriate, data in percentages and numbers are presented. The Chi-squared test was used to analyse the differences between categorical data. Fisher's exact test was used if the expected frequency was ≤5 in any cell. An independent samples t-test or the Mann Whitney U test were used to compare continuous variables depending on the normality of the distribution (as per the one-sample Kolmogorov-Smirnov test). A value of P<0.05 was considered to indicate a statistically significant difference.

## Results

### Patients enrolled and patient characteristics

The present study enrolled 25 patients with UC in remission and 51 patients with ASUC.

*UC cases in remission*. Among the 25 patients with UC in remission, 12 (48%) were male and the median age of the patients was 35 years (range, 22-60 years). The majority of the patients were in socioeconomic upper lower class (n=12) and lower middle class (n=8), as per the modified Kuppuswamy socieoeconomic scale (10). All the patients were in clinical remission for varying periods of time (3-96 months; mean, 19±24 months). E3 disease was the most common (10 patients, 40%) and no patient had any extraintestinal manifestations. The majority of the patients were on a combination of 5-ASA with thiopurine (16 patients, 64%) while the remainder were controlled on 5-ASA compounds alone (Table I).

*Patients with flares*. Among the 51 patients with ASUC included in the present study, 27 (53%) patients were male and the median age of the patients was 38 years (range, 16-65 years). The majority of the patients belonged to class II (upper lower) of the modified Kuppuswamy socioeconomic scale. The majority of the patient had extensive colitis (E3) (21 patients, 41%), whereas extra-intestinal manifestations (EIM) were observed in 11 (22%) patients, the most common being musculoskeletal manifestations (7 patients, 14%) (Table I). Of note, 31 (61%) of the patients were on 5-ASA compounds before the episode of flares, while 8 (16%) patients were on a combination of 5-ASA and azathioprine, and remainder of the patients were not on any form of therapy or had terminated treatment. While the condition of the majority of the treated patients improved with medical management (86%), 14% of the patients (7/51 patients) required surgery.

### Cost analysis. UC cases in remission

**Figure 1:**
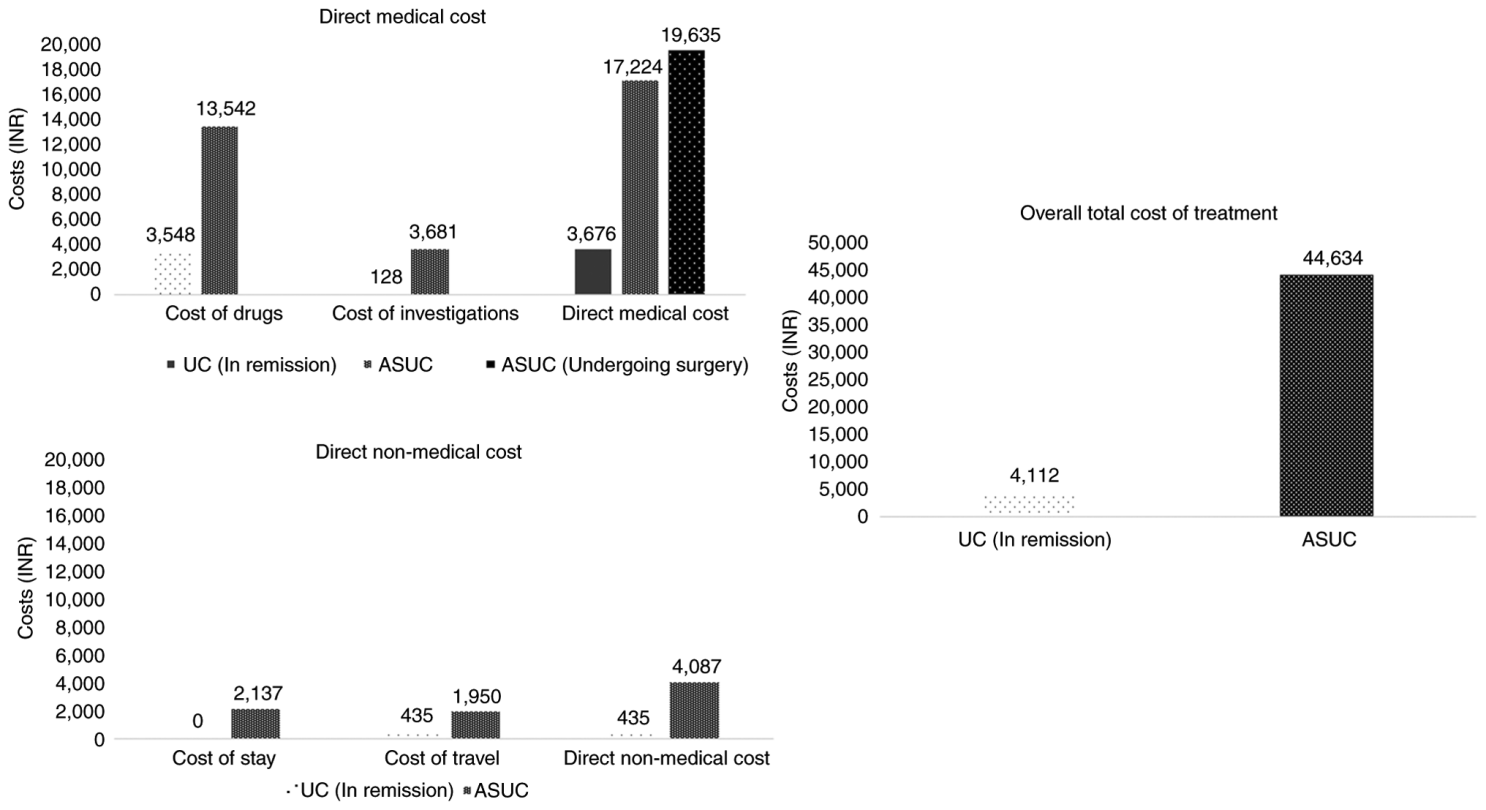
Cost of care for patients with ulcerative colitis in remission compared to patients with flares (acute severe ulcerative colitis). INR, Indian rupee.

Cost analysis of the patients with UC in remission revealed the average monthly expense due to direct medical costs to be 3,676/- INR, which included the cost of drugs (3,548/- INR), as well as investigations (128/- INR). The average direct non-medical cost was 435/- INR, which included only the travelling cost (435/- INR), as none of the patients in remission incurred additional expenses on accommodation during their OPD visits (Fig. 1). The average additional indirect cost incurred by the patients due to lost job earnings during the treatment period was negligible, as the majority of the patients could adjust their leave periods for planned hospital visits and did not lose any daily wages. Overall, the total per capita average monthly cost of treatment for patients with UC in remission in a tertiary government setup was calculated to be 4,112/- INR (Table II).

*Patients with flares*. For the management of an episode of ASUC, the average direct medical cost was 20,038/- INR, which included the cost of medical and surgical management (16,357/- INR), as well as cost of investigations (3,681/- INR). An additional per patient cost of 20,500/- INR incurred among patients undergoing surgery for the management of cases of ASUC. The average direct non-medical cost was 4,087/- INR, including the cost of stay during hospitalisation (2,137/- INR) and travelling costs (1,950/- INR). The average additional indirect costs incurred by the patients and their attendants due to lost job earnings during the treatment period was 20,509/- INR. Overall, the total per capita average cost of treatment for an episode of ASUC in a tertiary government setup was calculated to be 44,634/- INR (Table II). There was a significant difference (P<0.001) in the total cost of therapy among those who were managed conservatively with medical therapy alone vs. those who underwent surgical management. The total cost of therapy for the medically managed cases (n=44/51 patients, 86%) was estimated to be 19,615/- INR (15,150.5-27,034.5 INR), while the cost for those managed with surgical resection (n=7/51 patients, 14%) was 1,62,705/- INR (78,296-2,03,663 INR) (Table III).

The majority of the patients were admitted for 4-15 days in the hospital (86%), while those who underwent surgery had a significantly longer hospitalisation period (P=0.030). The median duration of patient admission was 8 days (IQR, 6-11 days). The median duration of hospital stays for patients managed with surgical resection (n=7/51 patients, 14%) was 14 days (IQR, 10.5-26), which was significantly longer (P=0.030) than the median duration of hospital stays for the patients who were managed only medical therapy alone (n=44/51 patients, 86%; median days of hospital stay, 7 days; IQR, 5.5-10.5) (Table III).

## Discussion

The present study demonstrated that the cost of managing patients with UC during flares is almost 11-fold the cost of managing patients with UC in remission, which highlights the importance of stringent disease control not only to decrease overall patient morbidity and improve quality of life, but also to lessen the overall financial burden. The cost of flares increases further if there is a need for surgical treatment. For patients with UC in remission, the direct medical cost (mostly contributed by the cost of drug) was responsible for almost 90% of the total cost of care, while the direct non-medical cost due to stay and travel during patient OPD follow-up was almost 10%; the indirect cost was negligible. In patients with acute flares with ASUC, the direct non-medical cost was ~10%. However, the direct medical cost due to drugs, surgery and investigations and the indirect cost due to the loss of wages almost equally contributed (~45% each) to the cost of illness.

Previous studies from Asia highlight similar trends of cost-of-illness in patients with UC. Kamat *et al* (11) computed the annual median cost of patients with UC in remission and relapse in southern India to be 43,140/- and 52,436.5/- INR, roughly translating into monthly cost of 3,595/- and 4,370/- INR, respectively. Direct costs of UC management were 84% compared to almost 100% observed in the patients with UC in remission in the present study (9). The recent study from Iran by Pakdin *et al* (12) estimated the mean annual costs for patients with UC to be US$ 1,077/- (~7,514/- INR per month). In direct medical costs, the cost of medication contributed to the majority of expenses (32%), while overall indirect costs due to both short-term and long-term disability contributed to major costs per patient (58%) among UC cases (12). The high financial impact resulting from short-term disability due to the temporary absenteeism of patients with ASUC and their caregivers was also a major determinant of cost-of-illness in the present study (45.95%).

The annual health care costs for patients with UC in remission in India are still lower than those estimated in western countries. The cost analysis of European IBD Inception cohort's 10 years follow-up evaluation revealed the annual per patient expenditure for patients with UC to be €1524/- (~11,878/- INR per month); among which the most costly contributions were due to medical and surgical hospitalisation (45%) (13). In the USA, the mean annual cost per patient with UC was calculated to be US$ 5,066/- (~35,343/- INR per month) (14). However, in the present study, none of the included subjects among the UC with remission group were maintained on newer advanced therapies, which could have underestimated the financial impact of maintenance therapy.

Flares of IBD negatively affect the quality of life of patients with UC. The severity of the flares has a direct bearing on the financial burden inflicted by the disease. Bassi (15), in a study conducted in the UK, demonstrated that when compared with quiescent cases of IBD, disease relapse was associated with a 2-3-fold increase in costs for non-hospitalised cases and a 20-fold increase in costs for hospitalised cases (15). In the present study as well, disease flares increased the cost of care of treatment by 11-fold and highlighted role of hospitalisation in major direct cost of care. The cost of flare requiring hospitalisation was estimated in a recent systematic review and meta-analysis across various continents for patients with UC (16). This ranges from US$ 187/- (~15,655/- INR) in Asia to US$ 3,874/- (~324,326/- INR) in the USA. In Europe, the mean cost of in-hospital care is US$ 1,236/- (~103,476/- INR) compared to 44,634/- INR in the present study (16).

The presents study demonstrated that the maximum financial burden in the care of patients with UC during periods of exacerbation was due to the lost productivity and absenteeism of patients and their caregivers, which translates into an indirect cost of care. Studies from western countries have highlighted similar results; for patients with UC belonging to the working-age population, the indirect cost burden may be greater than the direct cost burden to the patient (17,18). These findings highlight the need for the financial security system to tide over the state of acute financial crises during the period of hospitalisation for patients with acute UC flares. Other than stringent measures to control disease activity, social awareness of the disease and the collective effort of society to support for patients with IBD is required; this also emphasizes the need for health insurance, IBD patient support groups and financial support schemes for patients with IBD. Country-specific costs of medication differ and the inclusion of specific drugs in government or private insurance schemes empowers patients with their right to healthcare.

The are some limitations to the present study which should be mentioned. There is a possibility of a selection bias due to the inclusion of only a government tertiary referral centre in north India; this may have led to the underestimation of the costs due to the disproportionate representation of different social classes when compared to a private setup. Furthermore, none of the patients in remission was on advanced therapy or had undergone surgery in the past, which thus limits cost analysis to these subsets of patients. The costs of travel were standardised as per the distance from the hospital for public transport and could have underestimated the actual costs if the patients used private transport for their comfort and ease. In addition, the study may not have addressed the issues and costs to the caregivers who participate in the care of patients with flares.

However, the findings of the present study may prove helpful to treating physicians and may aid in the identification of the cost of managing flares. The findings presented herein are also a reminder that the cost of flares is not only limited to morbidity and mortality, but is also financial. To be on continuous treatment with maintenance therapy is also a financially prudent choice. It should also be noted that a large number of patients with flares were not on therapy, having terminated therapy with the improvement of symptoms.

## Figures and Tables

**Table I:** Demographic and clinical features of patients with UC in remission vs. those with flares (with ASUC).

Baseline clinical characteristics	Patients with UC in remission (n=25)	Patients with ASUC (n=51)	P-value
Age, median; (range) years	35 (22-60)	51 (16-65)	0.521
Male/female	12 (48%)/13 (52%)	27 (53%)/24 (47%)	0.684
SCCAI score; median (range)	0 (0-2)	11 (6-14)	<0.001
Disease free period	19±24 months	NA	
Extent of disease^a^			
E1(Y/N)	3 (12%)/22 (88%)	1 (2%)/50 (98%)	0.101
E2 (Y/N)	8 (32%)/17 (68%)	8 (16%)/43 (84%)	0.112
E3 (Y/N)	10 (40%)/15 (60%)	21 (41%)/30 (59%)	0.934
Unknown (Y/N)	4 (16%)/21 (84%)	21 (41%)/30 (59%)	0.038
Extraintestinal manifestations (Y/N)	0 (0%)/25 (100%)	11 (22%)/40 (78%)	0.013
Musculoskeletal	-	7 (14%)	
Ankylosing spondylitis	-	1 (2%)	
Aphthous ulcer	-	1 (2%)	
Pyoderma gangrenosum	-	1 (2%)	
Uveitis	-	1 (2%)	
Current therapy^a^			
None (Y/N)	0 (0%)/25 (100%)	12 (24%)/39 (76%)	0.007
5-ASA only (Y/N)	9 (36%)/16 (64%)	31 (61%)/20 (39%)	0.042
5-ASA with thiopurine (Y/N)	16 (64%)/9 (36%)	8 (16%)/43 (84%)	<0.001
Advanced therapy (Y/N)	0 (0%)/25 (100%)	0 (0%)/51 (100%)	-
Management			
Medical only	25 (100%)	44 (86%)	0.050
Surgical intervention	-	7 (14%)	
Outcome			
Recovered on medical therapy alone Y/N	25 (100%)/0(0%)	44 (86%)/7 (14%)	0.050
Underwent surgery and recovered	-	4 (8%)	
Underwent surgery but did not survive	-	3 (6%)	
Duration of hospital stay; median (range) days	NA	8 (2-46)	
<4 days	-	2 (4%)	
4-5 days	-	44 (86%)	
>15 days	-	5 (10%)	

Data are presented as number and percentage or as indicated. P-values were estimated using the Chi-squared test, Fisher's exact test or Mann-Whitney U-test.

^a^The comparisons made were with respect to the other patients without that parameter. NA, not applicable; UC, ulcerative colitis; ASUC, acute severe ulcerative colitis; Y, yes; N, no; 5-ASA, 5-aminosalicylic acid.

**Table II:** Cost of care for patients with UC in remission compared to patients with ASUC.

Cost-of-illness	Patients with UC in remission (n=25)	Patients with ASUC (n=51)
Direct medical cost	3,676/- (89.40%)	20,038/- (44.89%)
Cost of drugs	3,548/- (86.28%)	16,357/- (36.65%)
Cost of investigations	128/- (3.12%)	3,681/- (8.24%)
Direct non-medical cost	435/- (10.58%)	4,087/- (9.16%)
Cost of stay	0/- (0%)	2,137/- (4.79%)
Cost of travel	435/- (10.58%)	1,950/- (4.37%)
Indirect cost	1/- (0.02%)	20,509/- (45.95%)
Total cost	4,112/-	44,634/-

Costs are presented in INR. INR, Indian rupee; UC, ulcerative colitis; ASUC, acute severe ulcerative colitis.

**Table III:** Duration of hospital stay and cost-of-treatment among patients with ASUC (n=51) managed medically vs. surgically.

Hospital stay/costs	Patients with ASUC managed medically (n=44)	Patients with ASUC managed surgically (n=7)	P-value
Duration of hospital stay, days (IQR)	7 (5.5-10.5)	14 (10.5-26)	0.030
Cost of treatment (IQR)	19,615/- (15,150.50-27,034.50)	162,705/- (78,296-203,663)	<0.001

P-values were estimated using the Mann-Whitney U-test. Costs are presented in INR. INR, Indian rupee; ASUC, acute severe ulcerative colitis.

## Data Availability

The data generated in the present study may be requested from the corresponding author.
